# Excitation of coherent second sound waves in a dense magnon gas

**DOI:** 10.1038/s41598-019-44956-z

**Published:** 2019-06-21

**Authors:** V. Tiberkevich, I. V. Borisenko, P. Nowik-Boltyk, V. E. Demidov, A. B. Rinkevich, S. O. Demokritov, A. N. Slavin

**Affiliations:** 1 Department of Physics, OaklandUniversity, Rochester, MI USA; 2 Departmentof Physics and Center for Nonlinear Science, University of Muenster, Corrensstrasse 2-4, 48149 Muenster, Germany; 3 Kotel’nikov Institute of Radio Engineering and Electronics, RussianAcademy of Sciences, 125009 Moscow, Russia; 4 Institute of Metal Physics, Ural Division of RAS, Ekaterinburg, 620041 Russia

**Keywords:** Magnetic properties and materials, Bose-Einstein condensates, Spintronics

## Abstract

Second sound is a quantum mechanical effect manifesting itself as a wave-like (in contrast with diffusion) heat transfer, or energy propagation, in a gas of quasi-particles. So far, this phenomenon has been observed only in an equilibrium gas of *phonons* existing in liquid/solid helium, or in dielectric crystals (Bi, NaF) at low temperatures. Here, we report observation of a *room-temperature magnonic second sound*, or a wave-like transport of both energy and *spin angular momentum*, in a quasi-equilibrium gas of magnons undergoing Bose-Einstein condensation (BEC) in a ferrite film. Due to the contact of the magnon gas with pumping photons and phonons, dispersion of the *magnonic* second sound differ qualitatively from the phononic case, as there is no diffusion regime, and the second sound velocity remains finite at low wavenumbers. Formation of BEC in the gas of magnons modifies the second sound properties by creating an additional channel of energy relaxation.

## Introduction

If normal (or “first”) sound is a wave of density of molecules in a substance, the “second” sound is a wave of density of collective quasiparticle excitations that can be excited in the same substance^1^. Usually, second sound is discussed as a phenomenon in which energy transfer occurs in a wave-like fashion (in contrast with the usual diffusion-like heat transfer) in an equilibrium gas of *phonon* quasi-particles^2^. Second sound has been observed in a superfluid liquid He^4^ ^3–5^, in solid He^4^ ^6^, and in some dielectric solids, such as Bi^7^ or NaF^8^ at low temperatures. The condition for the observation of this interesting phenomenon is the requirement that gas of phonons exists in a state of thermal quasi-equilibrium, and the intensity of the so-called “umklapp” phonon-phonon scattering processes, that are responsible for the relaxation of the phonon momentum into a crystal lattice, is small^9–12^.

It should be, also, noticed, that in most experiments the second sound was excited by heat pulses, received using a resonant cavity^3,13^, and additional measures were necessary to prevent simultaneous excitation of both the first and the second sound^14^ in a phonon gas having a gapless spectrum.

In all the previous theoretical studies of the second sound phenomenon it was implicitly assumed that the system of quasi-particles carrying the sound waves is perfectly thermally isolated from the rest of the world. This assumption works well for phonons, which are responsible for the dominant part of the heat capacity of a solid or liquid, and for which heat (or energy) exchange with other subsystems can be ignored. The same, however, is not true for almost any other type of quasi-particles. Therefore, it is important to clarify how the heat exchange between the quasi-particle gas and other subsystems influences the properties of the second sound.

A particular example of such a “non-isolated” quasi-particle system is a dense quasi-equilibrium gas of *parametrically pumped* low-energy (microwave-frequency) magnons existing in high-quality yttrium-iron garnet (YIG) films^15–20^. The low-energy magnons exchange energy not only with the pumping photons, but also with both the lattice and higher-energy magnon branches of the YIG film spin system. This energy exchange leads to the formation of the room-temperature magnon Bose-Einstein condensate (BEC)^15–17^, and is responsible for the unusual dynamics of the magnon gas^18^. The dense gas of magnons is also an excellent model system for which many interesting novel phenomena, such as formation of quantized BEC vortices^21^, supercurrent flows in a magnonic BEC^21–24^, formation of hybrid magnetoelastic bosons^25^, and spin superfluidity^26,27^ have been either experimentally observed or theoretically studied recently.

The other peculiarity of magnons is the presence of a gap in the quasi-particle spectrum. The gap is proportional to the magnitude of the bias magnetic field, and, typically, is of the order of a few GHz. This allows one to excite the magnonic second sound *coherently*, by an alternating magnetic field of a sufficiently low frequency lying inside the spectral gap, which makes this signal incapable of excitation of regular magnons (magnonic “first” sound).

Besides, from a less general point of view of modern spintronics, the magnonic second sound would be rather interesting, because the propagating magnonic second sound transfers not only the energy (heat) but, also, the spin angular momentum. Thus, the magnonic second sound wave represents a *novel type of a spin current*, that is neither purely ballistic (carried by regular magnons)^28,29^, nor diffusive^29^, and, also, rather different from the magnonic supercurrents manifesting themselves in a room temperature BEC of magnons^21–24^.

Finally, an additional advantage of an externally pumped quasi-equilibrium magnon gas for the second sound experiments is the fact that by varying the pumping power one can change the magnon gas density, forcing it to undergo BEC, which allows one to study the influence of the BEC formation on the properties of the magnonic second sound waves.

## Experiment

**Figure 1:**
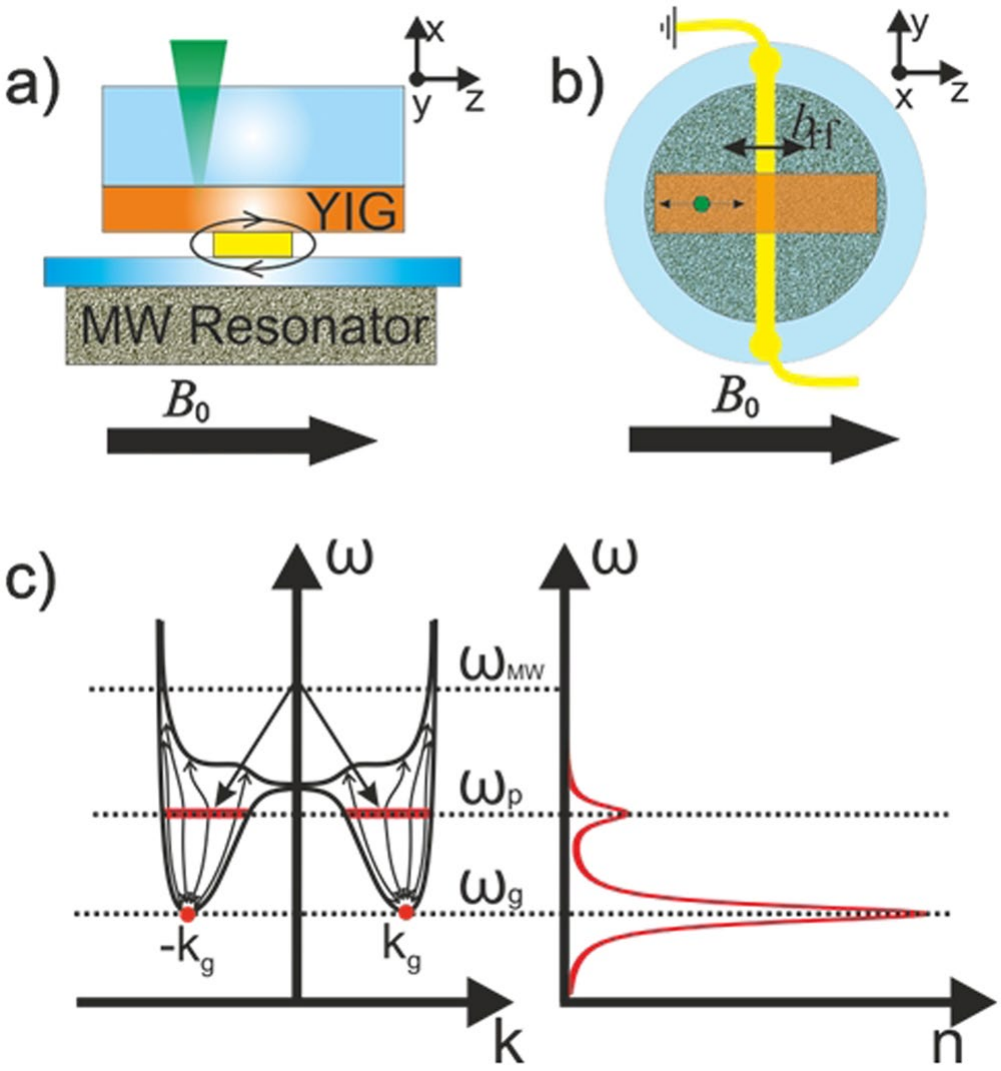
Experimental setup, frequency spectrum of magnons and spectrum of the measured Brillounin light scattering (BLS) signal: (**a**) Side view and (**b**) top view of the experimental setup. The YIG film magnetized in plane by a magnetic field *B*_0_ was attached to a dielectric resonator used to pump magnons. The magnonic second sound waves in the magnon gas were excited by a spatially non-uniform radio-frequency magnetic field, *b*_rf_, created by a gold conductor. A laser beam was focused on the YIG film and swept along the axis perpendicular to the conductor to obtain the magnon density at different distances from the conductor; (**c**) Frequency spectrum of magnons in a YIG film (left frame) and schematic view of the typical magnon spectrum measured by BLS. The simulated BLS-spectrum comprises two peaks: a peak with the frequency *ω*_p_/2π = *ω*_mw_/4π = 4.5 GHz corresponding to initial parametrically pumped magnons and a peak with the frequency *ω*_g_/2π = 3.8 GHz corresponding to the thermalized magnon gas near the bottom of the magnon spectrum at *k*_g_ and −*k*_g_.

A scheme of the experimental setup for the investigation of the second sound waves in a quasi-equilibrium magnon gas is shown in Fig. 1a,b. A dielectric resonator with the resonance frequency of *ω*_mw_/2π = *f*_mw_ = 9.0 GHz is used to inject magnons of a frequency *ω*_p_/2π =  *f*_p_ = *f*_mw_/2 = 4.5 GHz into a 5.1 μm thick in-plane magnetized YIG film by means of parametric pumping (see experimental details in^15,18^). Thermalization of the injected magnons, mainly caused by the four-magnon scattering^30^ results in the formation of a quasi-equilibrium magnon gas with a non-zero chemical potential, and also in the large overpopulation of the low-energy magnon states, eventually leading to the formation of BEC at room temperature^15–18,30,31^. For investigation of the magnon density, proportional to the magnon population function, using Brillouin light scattering (BLS) spectroscopy (see below for details) the laser beam was focused on a particular spot of the YIG film. Figure 1c illustrates the frequency spectrum of the excited magnons in the YIG film (left frame), and gives a schematic view of the expected BLS-spectrum, demonstrating the magnon population function (right frame). The setup was placed in the static magnetic field *B*_0_, which was equal to 133 mT for the most of the experiments.

The second sound waves in the thermalized magnon gas were coherently excited by a spatially non-uniform radio-frequency magnetic field, *b*_rf_, of the frequency *ω*_rf_/2π = *f*_rf_ = 0.5–10 MHz, created by a long, stripe-shaped conductor, as indicated in Fig. 1a,b. This excitation frequency was inside the spectral gap, well below the minimum frequency of regular magnons, that is *ω*_g_/2π = *f*_g_ ≈ 3.8 GHz at *B*_0_ = 133 mT. The conductor was aligned perpendicular to the static field, providing *b*_rf_ oriented parallel to *B*_0_. The resulting modulation of the total magnetic field acts as a time-dependent potential, causing an oscillation of the magnon density near the conductor, which, in turn, excites the waves of magnon density (magnonic second sound). Due to a large lateral aspect ratio of the stripe the excited second sound waves were quasi-one-dimensional with the propagation direction being perpendicular to the conductor, i.e., parallel to *B*_0_ (or to the *z*-direction in Fig. 1a) (see Methods for experimental details).

**Figure 2:**
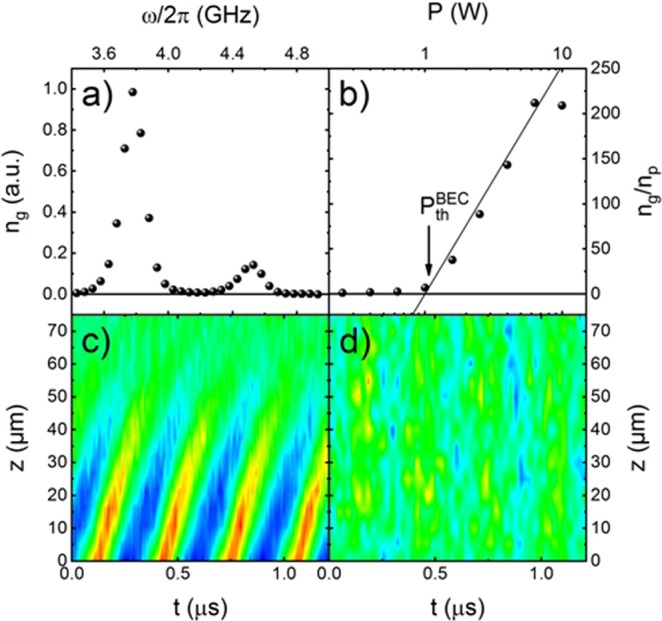
Experimental evidence of the “magnonic second sound” obtained using the BLS spectroscopy of the pumped quasi-equilibrium magnon gas near the bottom of the magnon spectrum: (**a**) Experimentally measured BLS spectrum, comprising peaks corresponding to the pumped magnons (*ω*_p_/2π = 4.5 GHz) and thermalized gas of magnons (*ω*_g_/2π = 3.8 GHz) (cf. Fig. 1c); (**b**) Ratio of intensities of the BLS peaks shown in (**a**) as a function of the pumping power; (**c**,**d**) Spatio-temporal map (*t* – time from the start of the rf-signal, *z* – the distance from the conductor) of the measured BLS intensity with red/blue color corresponding to the highest/lowest intensity. (**c**) – the map obtained for the thermalized magnon gas near the magnon spectral minimum (*ω* = *ω*_g_) demonstrates coherent excitation of the magnonic second sound wave by a radio-frequency driving signal of the frequency *f*_rf_ = 3 MHz at the pumping power *P*_*p*_ = 10 W; (**d**) – the map obtained for the primary magnons (*ω* = *ω*_p_) under the same driving and pumping conditions as (c). No propagating second sound waves are seen.

The magnon density, proportional to the magnon population function, was experimentally probed by Brillouin light scattering (BLS) spectrometry^32,33^ as illustrated in Fig. 1a,b. The corresponding BLS spectrum obtained in the YIG film at *B*_0_ = 133mT under the action of a microwave pumping with the power of *P*_*p*_ = 0.25 W and frequency *f*_mw_ = 9.0 GHz, is shown in Fig. 2a. In agreement with Fig. 1c, two distinct peaks are seen in the spectrum: a relatively weak peak at *f*_p_ = *f*_mw_/2 = 4.5 GHz, corresponding to the primary magnons created in the YIG film by the microwave pumping, and a substantially more intensive peak at the frequency *f*_g_ = 3.8 GHz caused by the quasi-equilibrium, thermalized magnons^30^ accumulated in the states existing close to the minima (corresponding to *k*_g_ and –*k*_g_) of the magnon spectrum (magnon ground state) of the film. The intensity of each of these two peaks is proportional to the density of magnons near the corresponding frequency. By varying the pumping power one can control the relative density of the accumulated magnons at different frequencies, as illustrated in Fig. 2b, where the ratio between the amplitudes of the two above mentioned BLS peaks is plotted versus the pumping power. One clearly sees that with the increase of the pumping power above 1 W the ratio between the number of magnons thermalized near the magnon ground state and the number of primary magnons increases substantially. As demonstrated by a direct observation of the interference of two magnon condensates in the real space^21^ and explained theoretically^17^, the point of the drastic growth of the density of the ground-state magnons marks the onset (or threshold) of the Bose-Einstein condensation of magnons.

By sweeping the laser beam away from the conductor, and using the time-resolved BLS spectroscopy^32,33^, we obtained spatio-temporal maps of the density of ground-state magnons (*ω* = *ω*_g_) and of primary magnons created by the microwave pumping (*ω* = *ω*_p_) when a driving radio-frequency signal was supplied. The examples of such maps obtained for the driving frequency *ω*_rf_/2π = *f*_rf_ = 3 MHz and pumping power *P*_*p*_ = 10 W at *B*_0_ = 133 mT are presented in Fig. 2c,d, where the color code reflects the magnon density, with red/blue color corresponding to the highest/lowest density.

It is clear from Fig. 2c,d, that the behavior of the quasi-equilibrium magnon gas thermalized near the ground-state (*ω* = *ω*_g_) and the primary pumped strongly non-equilibrium magnons (*ω* = *ω*_p_) under the influence of a periodic driving signal is drastically different. In the gas of ground-state quasi-equilibrium magnons the rf-field excites *propagating waves of the magnon density* with the slope of the lines of the constant density in Fig. 2c being proportional to the phase velocity of the waves. In contrast, in the ensemble of primary non-equilibrium magnons one observes just a weak periodic modulation of the magnon density, but no signature of propagating waves (see Fig. 2d).

**Figure 3:**
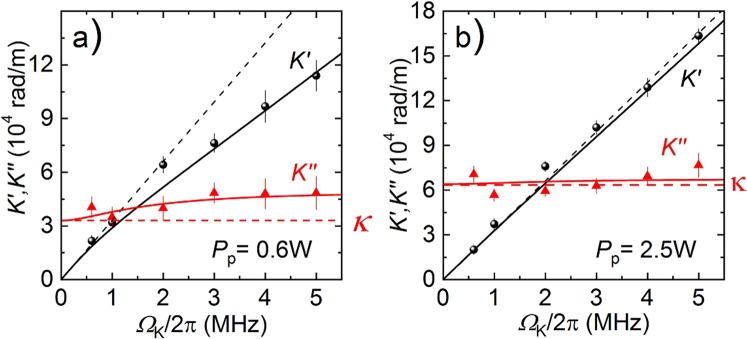
Dispersion of the magnonic second sound at two values of the pumping power (**a**) below (*P*_*p*_ = 0.5 W) and (**b**) above (*P*_*p*_ = 2.5 W) the BEC transition (*P*_th_^BEC^ = 1 W). Black and red symbols are the experimental data for the real and imaginary parts of the magnonic sound wavenumber. Solid lines are the best theoretical fits using Eqs (3), (4). Black dashed lines show the linear dispersion with constant sound velocity.

By analyzing the spatio-temporal maps similar to that shown in Fig. 2c we obtained the main parameters of the observed propagating waves of the density: its real and imaginary part of the complex wavenumber *K* = *K*′ + *iK*′′ at different frequencies. Each entire map for a given frequency of the sound wave, representing the time and space dependence of the wave amplitude was fitted by the following ansatz: ${\rm{\Delta }}n=A\exp (iK^{\prime} z-i{\Omega }_{K}t-{\phi }_{0})\exp (-K^{\prime\prime} z)$, where *A* is an overall scaling parameter and *φ*_0_ defines the origin of the time-scale. The dependences *K*′(*Ω*_K_) and *K*′′(*Ω*_K_) obtained from these fits are presented in Fig. 3. This figure shows how the fitted real *K*′ (black circles) and imaginary *K*′′ (red triangles) parts of the wavevector depend on the excitation frequency Ω_*K*_ for two values of the pumping power *P*_*p*_ (i.e., for two different magnon gas densities).

## Theory

A consistent theoretical description of the second sound waves in a gas of any quasiparticles, having the de Broglie wavevector ***k*** and frequency *ω*_***k***_, can be developed using a position- and time-dependent distribution function (quasi-particle population number) *n*_***k***_ ≡ *n*_***k***_(*t*,***r***), which obeys the Boltzmann equation^34,35^:

1 $$\frac{\partial {n}_{{\boldsymbol{k}}}}{\partial t}+{{\boldsymbol{v}}}_{{\boldsymbol{k}}}\cdot \nabla {n}_{{\boldsymbol{k}}}=S{t}_{{\boldsymbol{k}}}+{G}_{{\boldsymbol{k}}},$$

where ***v***_***k***_ = *∂ω*_***k***_/∂***k*** is the quasi-particle group velocity, *ω*_***k***_ is the quasi-particle dispersion relation, *St*_***k***_ is the collision integral that describes interactions of quasi-particles between themselves, and *G*_***k***_ is the term describing the interaction of quasi-particles with the other parts of the physical system. The dispersion relation Ω_*K*_(*K*′ + *iK*′′) of a second sound wave propagating in the gas of quasi-particles can be derived from Eq. (1), assuming the position-dependent thermal equilibrium (Bose-Einstein) distribution for the quasi-particle population number *n*_***k***_, and considering the dynamics of such integral quantities, as the quasi-particle population number $n=\int {n}_{{\boldsymbol{k}}}{d}^{3}{\boldsymbol{k}}/{(2\pi )}^{3}$, linear momentum ${\boldsymbol{p}}=\int \hslash {\boldsymbol{k}}\,{n}_{{\boldsymbol{k}}}{d}^{3}{\boldsymbol{k}}/{(2\pi )}^{3}$, and energy $e=\int \hslash {\omega }_{{\boldsymbol{k}}}\,{n}_{{\boldsymbol{k}}}{d}^{3}{\boldsymbol{k}}/{(2\pi )}^{3}$. These quantities are conserved in the inter-particle collisions, and, therefore, the contribution of the scattering integral *St*_***k***_ to their dynamics vanishes. As a result, in an isolated (*G*_***k***_ = 0) system the velocity of a second sound wave depends only on the quasiparticle dispersion relation *ω*_***k***_, and the thermodynamic properties of the gas distribution *n*_***k***_, and, in general, is close to the characteristic thermal velocity in the gas of quasi-particles.

In contrast to the case of an isolated gas of quasi-particles at the thermal equilibrium, in a quasi-particle gas existing in a state of *quasi-equilibrium* in contact with other subsystems, the exchange processes *G*_***k***_ could not be ignored. In a simplest model, these processes can be described as a linear relaxation of the integral quantities *n*, ***p***, and *e* towards their equilibrium “ambient” values. The relaxation rates for different quantities, in general, are different, and depend on the underlying physical scattering mechanisms.

Thus, in a situation involving the gas of phonons, as it was explained above, the heat exchange can be ignored, which means that the *energy relaxation rate is zero*. Typically, the only relevant process, describing interaction between photons and the other systems, is the relatively weak umklapp scattering, which is the exchange of a linear momentum between the phonons and the sample as a whole^9–12^. This process conserves the number of phonons, but changes their total linear momentum. Thus, for a phonon system the only significant relaxation process is the relaxation of its linear momentum. Due to this relaxation process the second sound in the gas of phonons becomes dissipative, and, in particular, in a low-frequency region the second sound in the presence of the umklapp scattering has a diffusive nature (conventional diffusive heat transfer).

In contrast, in a parametrically pumped gas of *magnons*, *all* the above mentioned integral quantities can change due to the interaction with the pumping, spin-lattice scattering, and interaction of low-energy magnons with higher-energy magnon branches^15,18,21,30,31^. Moreover, the intensity of these scattering processes, generally, increases with the increase of the magnon frequency *ω*_***k***_, which means that the energy (*e* ∝ *ω*_***k***_) relaxation rate Γ*e* should be larger than the relaxation rate Γ*p* of the linear momentum ($p\propto {\omega }_{{\boldsymbol{k}}}^{1/2}$).

Using the standard techniques of the theory of Boltzmann equation (see Supplementary Materials for details), and assuming arbitrary energy Γ*e* and momentum Γ*p* relaxation rates, we derived a general complex dispersion relation ${{\rm{\Omega }}}_{K}(K^{\prime} ,\,K^{\prime\prime} ,\,{{\rm{\Gamma }}}_{p},\,{{\rm{\Gamma }}}_{e})$ for the dissipative second sound of quasi-particles of an arbitrary nature:

2 $${{\rm{\Omega }}}_{K}^{2}+i({{\rm{\Gamma }}}_{p}+{{\rm{\Gamma }}}_{e}){{\rm{\Omega }}}_{K}+({{\rm{\Gamma }}}_{p}{{\rm{\Gamma }}}_{e}+{u}_{g}^{2}{K}^{2})=0,$$

where *K* = *K*′ + *iK*′′ is the complex wavenumber of the second sound wave, and *u*_*g*_ is the phase velocity of the second sound in the absence of relaxation. Note, that the corresponding dispersion equation for a *phononic* second sound follows from Eq. (2) in the limit Γ*e* = 0 (compare to, e.g., Eq. (29) in^11^).

The frequency Ω_*K*_ and the coefficient of spatial damping *K*′′ (imaginary part of the wavenumber) of the second sound wave are connected with the (real) wavenumber *K*′ by the simple expressions:

3 $$\begin{array}{cc}{{\rm{\Omega }}}_{K}={V}_{K}K^{\prime} , & K^{\prime\prime} =\kappa \frac{{V}_{K}}{{u}_{g}},\end{array}$$

where *V*_*K*_ is the phase velocity of the second sound

4 $${V}_{K}={u}_{g}\sqrt{\frac{{q}^{2}{\kappa }^{2}+{(K^{\prime} )}^{2}}{{\kappa }^{2}+{(K^{\prime} )}^{2}}},$$

$\kappa =({{\rm{\Gamma }}}_{p}+{{\rm{\Gamma }}}_{e})/(2{u}_{g})$ is the characteristic second sound damping parameter, $q=2\sqrt{{{\rm{\Gamma }}}_{p}{{\rm{\Gamma }}}_{e}}/({{\rm{\Gamma }}}_{p}+{{\rm{\Gamma }}}_{e})$ is the relaxation asymmetry parameter characterizing the difference between the relaxation rates Γ*p* and Γ*e* for the energy and linear momentumof quasi-particles, respectively, and

5 $${u}_{g}=\sqrt{\frac{10}{9}\frac{{E}_{av}}{m}}$$

is the dissipationless second sound velocity (in the absence of relaxation, *κ* = 0). In this expression *E*_*av*_ is the average quasi-particle energy measured from the minimum of the spectrum and *m* is the quasiparticle’s effective mass (see Supplementary Materials for details).

The dispersion equation for the case of an *ideal dissipationless* gas (like in the case of a superfluid liquid He^4^ ^3–5^) is obtained from Eq. (3) when *κ* = 0 and yields a simple expression with frequency-independent sound velocity Ω_K_/*K* = *V*_*K*_ = *u*_*g*_. In the case of a real gas of *phonons*, which are in a thermally-isolated thermodynamic equilibrium, but are affected by the “umklapp” phonon momentum relaxation processes (*κ* ≠ 0, but *q* = 0, which is the case of a solid He^4^ ^6^ and dielectric solids^7,8^), the second sound becomes dissipative, and demonstrates transition from wave-like to diffusion-like (*K*′′ ≈ *K*′) propagation below certain cutoff sound frequency.

**Figure 4:**
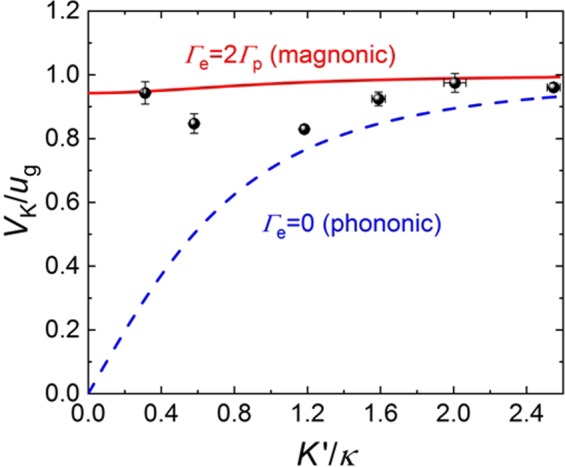
Phase velocities *V*_*K*_(*K*′) of the “magnonic” (*q* ≠ 0) and “phononic” (*q* = 0, *κ* ≠ 0) dissipative second sound waves as functions of the wavenumber *K*′. In the calculations of the “magnonic” dispersion characteristics it was assumed that Γ_*e*_ = 2Γ_*p*_. The experimental results for the magnonic second sound taken from Fig. 3b are shown by black dots.

In contrast to phonons, magnon gas is in a thermodynamic equilibrium with non-vanishing heat exchange (system with a heat contact, *κ* ≠ 0 and *q* ≠ 0). It follows from Eqs (3), (4) that the dispersion properties of such “*magnonic*” second sound are rather different from “*phononic*” case. While the sound velocity *V*_*K*_(*K*′) and the damping parameter *K*′′(*K*′) of the *phononic* second sound vanishes in the long-wave limit *K*′→0, demonstrating a typical diffusion-like behavior, the velocity and the damping parameter of the *magnonic* second sound remains practically constant with the variation of the wavenumber *K*′. Thus, the *magnonic* second sound demonstrates wave-like propagation (although with considerable damping) up to zero sound frequency. It is also clear, that the results of our experiments (shown by dots in Fig. 4) qualitatively agree with the “magnonic” (*q* ≠ 0) case, and do not agree at all with the “phononic” equations (*q* = 0) (see Fig. 4).

The relations Eq. (3) were used to fit the experimental dispersion curves Ω_*K*_(*K*′ + *iK*′′) for the magnonic second sound (similar to Fig. 3) obtained at different magnon densities with *κ* and *q* used as the fitting parameters dependent on the magnon density, and, therefore, on the pumping power. The fitting results are shown in Fig. 3a,b by solid lines and demonstrate good agreement with the experimental data. The dimensionless parameter *q*, which characterizes asymmetry of the momentum and energy relaxation, increases with pumping power from *q* ≈ 0.5 to *q* ≈ 0.5, while *κ* has a non-monotonic dependence on *P*_*p*_ reaching maximum value *κ* ≈ 6 × 10^4^ rad/cm at *P*_*p*_ = 2.5 W and gradually decreasing for both larger and smaller values of *P*_*p*_. The detailed dependence of the parameters *κ* and *q* on the pumping power *P*_*p*_, obtained from the fitting procedure, is shown in the Supplemental Materials (see Fig. S1).

## Second Sound and Bose-Einstein Condensation

The above developed theoretical framework Eqs (2–5) allows one to use the experimentally measured complex dispersion characteristics of the dissipative magnonic second sound as a probing tool for the investigation of the internal properties of the quasi-equilibrium pumped and damped gas of magnons formed near the bottom of the magnon spectrum. For that gas all the processes of the magnon-magnon interaction, redistributing the pumped magnons between the low-energy region near the spectral minimum and either the high-energy region of the magnon spectrum^18^ or the BEC of magnons formed exactly at the spectral minimum^15^, can be interpreted as the processes of linear relaxation of the energy and momentum of the magnons forming a low-energy quasi-equilibrium magnon gas. With the increase of the overall gas density, caused by the increase of the pumping power, the gas of magnons acquires a non-zero chemical potential, and these redistribution processes manifest themselves as the variation of the relaxation parameters Γ_*p*_ and Γ_*e*_ and the average energy of the magnons *E*_*av*_ = *e*_*g*_/*n*_*g*_ (see Supplemental Materials for details).

We would like to stress here one difference of the magnon system undergoing BEC transition with other bi-component gasses (e.g., ^4^He), which justifies the presented above simple picture of interaction between normal and BEC phases in a magnon gas. In a ^4^He system, the total density of particles is fixed, and, as a result, any fluctuation of the normal phase density is accompanied by the opposite fluctuation of the density of the BEC (superfluid) phase. Thus, the phonon excitations in ^4^He and similar bi-component systems equally involve both (normal and superfluid or BEC) phases, and the formation of a BEC strongly modifies the properties of the second sound in a gas of phonons.

In contrast, in a magnon gas the total magnon density is not fixed, and the fluctuations in the normal and BEC phases may occur independently. The experimental findings shown below agree well with our theoretical analysis based on this assumption, which proves that the magnonic second sound propagates mostly in the normal magnon phase, even after the formation of a BEC in the gas of magnons.

**Figure 5:**
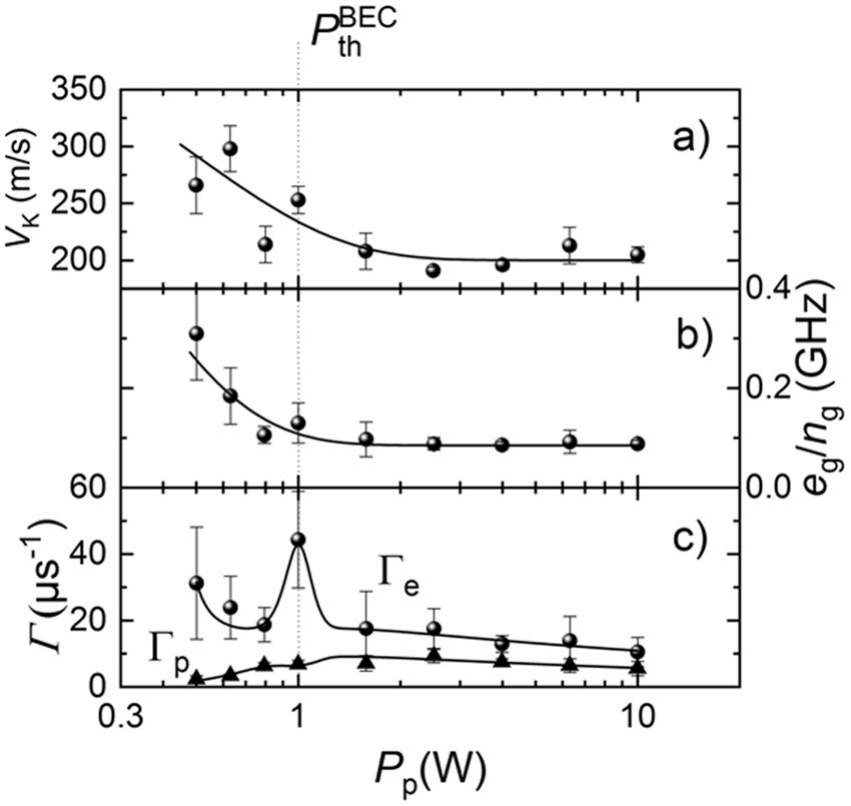
(**a**) Phase velocity *V*_*K*_ of the magnonic second sound, (**b**) averaged energy of the thermalized magnons *E*_*av*_ = *e*_*g*_/*n*_*g*_, and (**c**) linear relaxation parameters for the magnon energy Γ_*e*_ and magnon linear momentum Γ_*p*_ vs pumping power *P*_*p*_. The curves were calculated from the experimental dispersion curves of the magnonic second sound similar to those shown in Fig. 3 by fitting Eqs (3–5). Vertical dashed line shows the power threshold of the magnonic BEC formation (*P*_th_^BEC^ = 1 W) determined from Fig. 2b.

Figure 5 demonstrates the dependences of the (a) phase velocity *V*_*K*_ of the magnonic second sound, (b) averaged energy of the thermalized magnons *E*_*av*_ = *e*_*g*_/*n*_*g*_, and (c) linear relaxation parameters for the magnon energy Γ_*e*_ and magnon linear momentum Γ_*p*_ as functions of the pumping power (or density of the magnon gas). All these quantities were calculated from the experimental dispersion curves similar to those shown in Fig. 3 by fitting to Eqs (3–5) for each value of the pumping power. The vertical dashed line shows the power threshold of the BEC formation (*P*_th_^BEC^ = 1 W) determined from Fig. 2b (see^21^ for details).

First of all, Fig. 5 demonstrates that no dramatic changes of the dispersion properties of the magnonic second sound take place when the magnon gas undergoes a BEC transition. It can be seen from Fig. 5b, that the average magnon energy *E*_*av*_ = *e*_*g*_/*n*_*g*_ in the thermalized gas of low-energy magnons goes down with the increase of the magnon density up to the threshold of the BEC formation, above which it remains constant. This behavior is consistent with the theoretical model, which predicts that the average energy of the thermalized magnons should decrease with the increase of the chemical potential (see Supplementary Materials for details). The constant value of *E*_*av*_. above the BEC threshold gives additional independent proof that the chemical potential of the magnon gas reached its maximum value equal to the minimum energy in the magnon spectrum (see Eq. (S13) in the Supplementary Materials for details). At the same time, the relaxation parameter Γ_*e*_ of the magnon energy goes through a sharp maximum at the BEC threshold (Fig. 5c) showing that the BEC of magnons creates just an additional channel of relaxation for the magnonic second sound. Ts behavior is similar to the temperature dependences of the magnetic susceptibility of a paramagnet close to its ferromagnetic Curie temperature^36^ or that of the thermal capacitance of the liquid helium near the superfluid transition^37^. This characteristic feature can be considered as an additional experimental signature of the BEC transition in a gas of pumped magnons.

**Figure 6:**
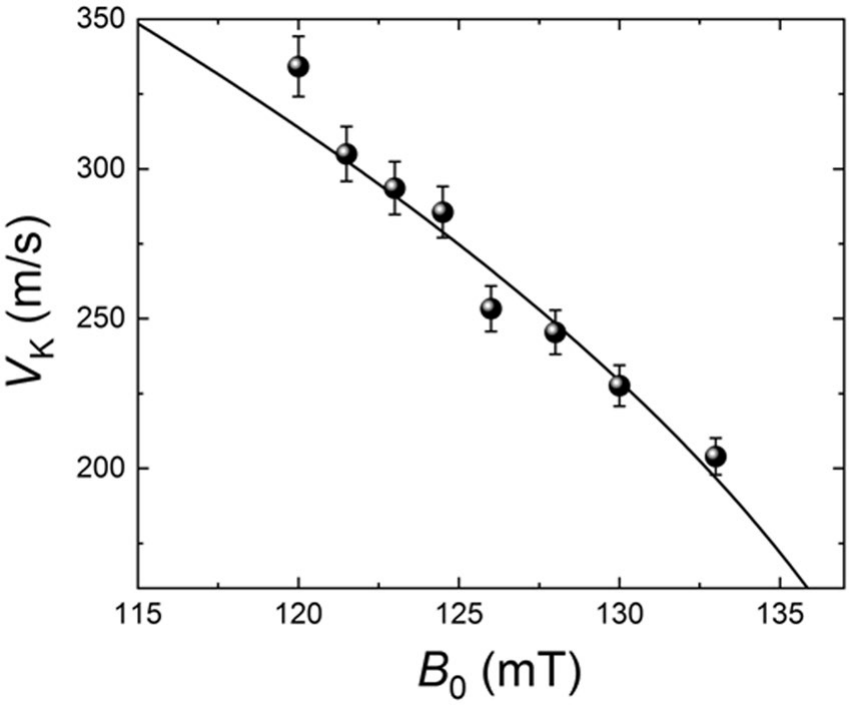
Phase velocity *V*_*K*_ of the magnonic second sound as a function of the bias magnetic field *B*_0_: Symbols – experimental values taken at *P*_*p*_ = 10.0 W and *f*_mw_ = 9.0 GHz, line – theoretical result calculated for *q* = 1 (see Fig. S1) from Eqs (4), (5) assuming that Δ*ω*_*th*_/2*π* = 490MHz (see Supplemetary Materials for details).

All the above data were collected at a constant static magnetic field *B*_0_ = 133 mT. To gain further insight into the above described picture of the coherent excitation of a magnonic second sound in a thermalized gas of parametrically pumped magnons we measured the dependence of the magnonic second sound velocity on the bias magnetic field *V*_*K*_(*B*_0_) at the pumping power *P*_*p*_ = 10 W, when the BEC of magnons is fully formed. The results of these measurements are presented in Fig. 6 in comparison with the theoretical curve calculated using Eqs (3), (4). It is clear, that the theory gives a good quantitative description of the experiment when the lowest energy of the magnon spectrum is varied with the variation of the bias magnetic field *B*_0_.

Although the development of a microscopic theory of the BEC of magnons in magnetic films under parametric pumping is necessary to understand all the details of the magnon-magnon interaction processes taking place in this system at large magnon densities, we believe, that the experimental discovery of the dissipative *magnonic second sound*, having dispersive properties dramatically different from that of a traditionally studied phononic second sound, is important, and creates a new tool to probe experimentally the internal properties of a quasi-equilibrium gas of magnons undergoing BEC at room temperature. We also believe, that the discovered properties of the magnonic second sound are general, and will be observed in a dense gas of quasi-particles of any physical nature if this gas is externally pumped, so that both energy and linear momentum of its particles undergo relaxation processes to the quasi-equilibrium values of these quantities.

## Methods

### Sample preparation and parameters

The sample with the lateral dimensions of the 2 × 5 mm was cut from a monocrystalline yttrium-iron-garnet (YIG) (111) film with the thickness of 5.1 µm grown on a gallium gadolinium garnet substrate. As shown in Fig. 1a,b, the sample was magnetized by a magnetic field *B*_0_ applied in the plane and was attached to a dielectric resonator with the resonant frequency *f*_mw_ = 9.0 GHz, used to pump magnons parametrically into YIG film. Due to thermalization of the primary pumped magnons a coherent condensate was created at the frequency, corresponding to the minimum of the magnon spectrum *f*_g_ (*f*_g_ = 3.8 GHz for *B*_0_ = 133 mT), if the density of the primary magnons was sufficiently high. The corresponding threshold pumping power was about 1 W. For lower pumping powers one obtains a quasi-equilibrium gas of incoherent magnons.

### Excitation of second sound

The “magnonic second sound” waves in the magnon gas were excited by a spatially non-uniform radio-frequency magnetic field, *b*_rf_, of the frequency *f*_rf_ = 0.5–10 MHz, which was generated by a gold conductor with a length of 6 mm and a width of 10 μm. The conductor was lithographically patterned from a gold film with a thickness of 300 nm sputtered on a sapphire plate. The orientation of the conductor was chosen in such a way that it produces a radio-frequency magnetic field parallel to *B*_0_. The value of *b*_rf_ close to the conductor was 6.25 mT, i.e., *b*_rf_ ≪ *B*_0_.

### Brillouin light scattering (BLS) spectroscopy

The magnetization dynamics in the YIG film was studied by means of space- and time-resolved Brillouin light scattering (BLS) spectroscopy^32,33^. The laser beam went through the transparent GGG substrate and was focused on YIG film. The spatial resolution of the recorded magnon density maps is mainly determined by the diameter of the beam focus which was about 5 μm, whereas the temporal resolution provided by the time-resolved BLS is about 1 ns. Since the BLS signal at a given frequency is proportional to the magnon density at this frequency, using this technique one can map the density waves (second sound) propagating in the magnon gas. For this purpose the sample together with the rf-conductor and mw-resonator was mounted on a translational mechanical-stage allowing positioning of the entire arrangement with respect to the probing light beam in the y-z plane with an accuracy of 1 μm.

## Supplementary information

Supplementary materials

## Data Availability

The data are available from the corresponding author upon request.
