# Supplementary material for: Excitation of coherent second sound waves in a dense magnon gas
Source: Sci Rep. 2019 Jun 21;9:9063. doi: 10.1038/s41598-019-44956-z (PMC6588561; doi:10.1038/s41598-019-44956-z)
Supplement: Supplementary file 1 — Supplementary materials [file 41598_2019_44956_MOESM1_ESM.docx]

**Supplementary materials for the manuscript**

**“Excitation of coherent second sound waves in a dense magnon gas”**

V. Tiberkevich^1^^[[1]](#footnote-2)^ , I. V. Borisenko^2,3^, P. Nowik-Boltyk^2^, V. E. Demidov^2^ , A.B. Rinkevich^4^,
S. O. Demokritov^2,4^ , and A.N. Slavin^1^

^1^*Department of Physics, Oakland University, Rochester, MI, USA*

^2^*Department* *of Physics and Center for Nonlinear Science, University of Muenster, Corrensstrasse 2-4, 48149 Muenster, Germany*

*^3^Kotel’nikov Institute of Radio Engineering and Electronics, Russian Academy of Sciences, 125009 Moscow, Russia*

^4^*Institute of Metal Physics, Ural Division of RAS, Ekaterinburg 620041, Russia*

To analyze the problem of the second sound propagation in a gas of quasi-particles (e.g., magnons) existing in a heat contact with other degrees of freedom of a physical system, we start from the Boltzmann equation (1) for the distribution function (particles’ population number) $n_{\boldsymbol{k}}=n_{\boldsymbol{k}}\left( t,\boldsymbol{r} \right)$ of gas particles [S1]:

$\frac{\partial n_{\boldsymbol{k}}}{\partial t}+\boldsymbol{v}_{\boldsymbol{k}}\cdot\nabla n_{\boldsymbol{k}}=St_{\boldsymbol{k}}+G_{\boldsymbol{k}}$, (S.1)

Here $\boldsymbol{v}_{\boldsymbol{k}}={\partial\omega_{\boldsymbol{k}}}/{\partial\boldsymbol{k}}$ is the group velocity of a quasi-particle with a wavevector $\boldsymbol{k}$, $\omega_{\boldsymbol{k}}$ is the quasi-particle frequency, $St_{\boldsymbol{k}}$ is the usual collision integral that describes mutual quasi-particle interaction and is responsible for the thermalization processes, and $G_{\boldsymbol{k}}$ is the “extrinsic collision integral” describing interaction of quasi-particles with other degrees of freedom.

Quasi-particle gas can be considered as a separate thermodynamic subsystem as long as the intrinsic thermalization is much faster than the interaction with the “rest of the world”, i.e. when $\left| St_{\boldsymbol{k}} \right|\gg\left| G_{\boldsymbol{k}} \right|$. It should be noted, that, in the particular case that we studied experimentally – case of a dense parametrically-pumped magnon gas – the pumped quasi-equilibrium state is established only in a relatively small region of magnon energies close to the minimum of the magnon spectrum [S2] (approximately, from the minimum magnon energy to the energy of the parametrically injected magnons [S2-S4]). In this case, the “rest of the world” degrees of freedom include, also, the higher-energy magnon branches that lie outside the pumped quasi-equilibrium range. Due to the substantially increased magnon density in the pumped quasi-equilibrium region, the condition $\left| St_{\boldsymbol{k}} \right|\gg\left| G_{\boldsymbol{k}} \right|$ still holds in this case.

The leading term $St_{\boldsymbol{k}}$ in Eq. (S.1) describes the intrinsic scattering processes that drive the quasi-particle distribution $n_{\boldsymbol{k}}$ towards the *local* equilibrium Bose-Einstein distribution

$n_{\boldsymbol{k}}=\frac{1}{\exp\left[ \left( \hbar\omega_{\boldsymbol{k}}-\boldsymbol{U}\cdot\hbar\boldsymbol{k}-\mu\right)/{k_{B}T} \right]-1}$ (S.2)

with the thermodynamic parameters determined by the local densities of all the relevant conserved quantities. In the Eq.(S.2) $T$ is the effective thermodynamic temperature, $\mu$
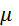
 is the chemical potential of quasi-particles, $k_{B}$ is the Boltzmann constant, and the vector parameter $\boldsymbol{U}$ denotes the characteristic quasi-particle velocity. Note, that since the propagation of the second sound waves in a gas of quasi-particles breaks the inversion symmetry of the quasi-particle distribution, the inclusion of a non-zero average velocity of quasi-particles $\boldsymbol{U}$ is necessary to describe the second sound waves.

As a first approximation, valid for the second sound waves of a relatively low frequency, one can assume that the distribution function of quasi-particles always has the form Eq. (S.2) with certain (position- and time-dependent) values of the thermodynamic parameters $k_{B}T$, $\mu$, and $\boldsymbol{U}$. This approach implicitly takes into account all the intrinsic scattering processes $St_{\boldsymbol{k}}$, which will not appear explicitly in the following analysis.

Instead of the thermodynamic parameters $k_{B}T$, $\mu$, and $\boldsymbol{U}$, it is more convenient to characterize the local quasi-particle equilibrium state using equivalent set of densities of conserved quantities, namely, density of energy $e=\int{\hbar\omega_{\boldsymbol{k}}n_{\boldsymbol{k}}d^{3}\boldsymbol{k}}/{\left( 2\pi\right)^{3}}$, density of particles $n=\int{n_{\boldsymbol{k}}d^{3}\boldsymbol{k}}/{\left( 2\pi\right)^{3}}$, and density of linear momentum $\boldsymbol{p}=\int{\hbar\boldsymbol{k} n_{\boldsymbol{k}}d^{3}\boldsymbol{k}}/{\left( 2\pi\right)^{3}}$, correspondingly. Since these are the integrals of motion of a closed physical system, the intrinsic gas dynamics may only lead to spatial redistribution of these quantities.

The peculiarities of the extrinsic relaxation processes described by the term $G_{\boldsymbol{k}}$, obviously, depend on the type of quasi-particle gas under consideration. It is possible, however, to devise a simple and quite general model of the extrinsic relaxation processes, that should be valid for any quasi-particle system in the case of low-amplitude and low-frequency second sound waves. Namely, when the quasi-particle distribution function does not differ significantly from its “global” equilibrium form, the “extrinsic” scattering processes can be described reasonably well as a linear relaxation of the quasi-particle gas towards a certain “global” equilibrium (which is dependent on the type of a quasi-particle gas). In other words, the “extrinsic” scattering processes $G_{\boldsymbol{k}}$ could be approximately described by the following phenomenological relaxation equations:

$\left( \frac{\partial n}{\partial t} \right)_{G}=-\Gamma_{n}\left( n-n_{g} \right)$, (S.3a)

$\left( \frac{\partial\boldsymbol{p}}{\partial t} \right)_{G}=-\Gamma_{p}\boldsymbol{p}$
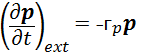
, (S.3b)

$\left( \frac{\partial e}{\partial t} \right)_{G}=-\Gamma_{e}\left( e-e_{g} \right)$
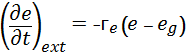
. (S.3c)

Here $n_{g}$ and $e_{g}$ are the equilibrium density of the quasi-particles and density of the quasi-particle energy, respectively, in the absence of a second sound wave. The equilibrium density of the quasi-particle linear momentum is assumed to be zero ($\boldsymbol{p}_{g}=0$). The parameters $\Gamma_{n}$, $\Gamma_{p}$, and $\Gamma_{e}$ are the relaxation rates of the corresponding conserved quantities, which depend on the equilibrium conditions (e.g., on the pumping power and frequency in the case of a pumped gas of magnons), and are, in general, different from each other. In particular, the usual second sound waves in a gas of phonons with only Umklapp processes are characterized by $\Gamma_{n}=\Gamma_{e}=0$ and $\Gamma_{p}\neq0$ [S5, S6].

In contrast, in the case of a *magnonic second sound* in a *pumped* gas of magnons, the dominant extrinsic relaxation processes are the interaction of magnons in the thermalized pumped region with the higher-energy magnons [S2]. Such processes conserve neither of the quantities $n$, $\boldsymbol{p}$, and $e$, and, therefore, in a pumped magnonic gas the three relaxation rates $\Gamma_{n}$, $\Gamma_{p}$, and $\Gamma_{e}$ could be different, but should have the same order of magnitude.

Equations (S.3) represent the simplest form of the relaxation equations, and several assumptions were made to derive these equations. For example, the “extrinsic” scattering in Eqs. (S.3) is considered to be a completely local and instantaneous process. This assumption may become incorrect in a short-wavelength (high-frequency) region of the second sound wave spectrum. Also, Eqs. (S.3) do not contain cross-relaxation terms, which are also possible in a linear approximation. In addition, the momentum relaxation process in Eqs. (S.3) is assumed to be isotropic, which may be incorrect for an anisotropic quasi-particle spectrum (this note, however, is irrelevant for the problem of a one-dimensional wave propagation).

Nonetheless, Eqs. (S.3) provide a fair approximate description of the real “extrinsic” quasi-particle scattering processes that could be given in the framework of a relatively simple and general theory.

To simplify the following analysis and to avoid the numerical calculation of the relevant integrals, we shall assume that the considered quasi-particles have a parabolic spectrum:

$\omega_{\boldsymbol{k}}\boldsymbol{=k}\cdot\hat{\boldsymbol{D}}\cdot\boldsymbol{k}$. (S.4)

Here $\hat{\boldsymbol{D}}$ is the dispersion matrix (diagonal matrix with the coefficients $D_{x}$, $D_{y}$, and $D_{z}$). The parabolic approximation is always valid in a vicinity of an energy minimum, and the dispersion matrix near this minimum can always be written in a diagonal form by a proper choice of coordinates.

For the parabolic dispersion Eq. (S.4), the wave group velocity is directly proportional to the wavevector $\boldsymbol{k}$,

$\boldsymbol{v}_{\boldsymbol{k}}=2\hat{\boldsymbol{D}}\boldsymbol{\cdot k}$
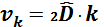
, (S.5)

and the equilibrium distribution Eq. (S.2) can be rewritten as:

$n_{\boldsymbol{k}}\boldsymbol{=}\frac{1}{\exp\left[ \left( \hbar\omega_{\boldsymbol{k-}\boldsymbol{k}_{0}}-\mu' \right)/{k_{B}T} \right]-1}$
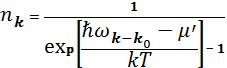
, (S.6)

where

$\boldsymbol{k}_{0}=\frac{1}{2}{\hat{\boldsymbol{D}}}^{-1}\cdot\boldsymbol{U}$
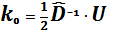
 (S.7a)

and

$\mu^{'}=\mu+\boldsymbol{k}_{0}\cdot\hat{\boldsymbol{D}}\cdot\boldsymbol{k}_{0}=\mu+\frac{1}{4}\boldsymbol{U}\cdot{\hat{\boldsymbol{D}}}^{-1}\cdot\boldsymbol{U}$
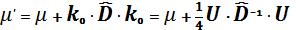
. (S.7b)

Thus, the effect of a non-zero average velocity $\boldsymbol{U}$ of quasi-particles is equivalent to a shift of the distribution function in the $\boldsymbol{k}$-space. Using this fact and the fact that the quasi-particle spectrum Eq. (S.4) is symmetric with respect to the inversion, one can simplify the integrals involving the distribution function $n_{\boldsymbol{k}}$. In particular:

$\int{\boldsymbol{v}_{\boldsymbol{k}}n_{\boldsymbol{k}}d^{3}\boldsymbol{k}}/{\left( 2\pi\right)^{3}=\frac{2}{\hbar}\hat{\boldsymbol{D}}\cdot\boldsymbol{p}}$
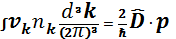
,

$\boldsymbol{p}=\int{\hbar\boldsymbol{k} n_{\boldsymbol{k}}d^{3}\boldsymbol{k}}/{\left( 2\pi\right)^{3}}=\hbar\boldsymbol{k}_{0}n$
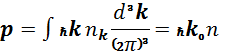


We would like to clarify, that the wavevector $\boldsymbol{k}_{0}$ and velocity parameter $\boldsymbol{U}$ are *not* related to the wavevector and speed of the second sound wave. The parameters $\boldsymbol{k}_{0}$ and $\boldsymbol{U}$ describe asymmetry of the local distribution function and are proportional to the amplitude of the second sound wave. In this respect the parameters $\boldsymbol{k}_{0}$ and $\boldsymbol{U}$ are similar to the *particle’s* velocity (as opposed to the *wave* velocity) in a conventional wave.

Now, one can derive a closed system of equations governing the dynamics of the conserved quantities during the propagation of a second sound wave in a quasi-particle gas. We shall consider here the propagation of a plane second sound wave in the $x$ direction, so that all the functions will depend only on the $x$ spatial coordinate, and the wave vector will have the form $\boldsymbol{k}_{0}=k_{0}\boldsymbol{e}_{x}$. Using all the above described simplifications in Eq. (S.1), one can derive the following equations:

$\frac{\partial n}{\partial t}+\frac{2D_{x}}{\hbar}\frac{\partial p_{x}}{\partial x}=-\Gamma_{n}\left( n-n_{g} \right)$, (S.8a)

$\frac{\partial p_{x}}{\partial t}+\frac{2}{3}\frac{\partial e}{\partial x}=-\Gamma_{p}p_{x}$, (S.8b)

$\frac{\partial e}{\partial t}+\frac{\partial}{\partial x}\left( \frac{10}{3}D_{x}k_{0}e+2\hbar D_{x}^{2}k_{0}^{3}n \right)=-\Gamma_{e}\left( e-e_{g} \right)$. (S.8c)

For a small-amplitude second sound waves the parameter $k_{0}$ is a small quantity in comparison with the width of the distribution function $n_{\boldsymbol{k}}$ in the $k$-space, which allows one to simplify Eq. (S.8c) to the following form:

$\frac{\partial e}{\partial t}+\frac{3}{2}u_{g}^{2}\frac{\partial p_{x}}{\partial x}=-\Gamma_{e}\left( e-e_{g} \right)$
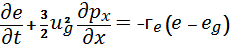
. (S.8c′)

Here

$u_{g}^{2}=\frac{20D_{x}E_{av}}{9\hbar}=\frac{10}{9}\frac{E_{av}}{m}$
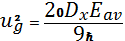
 (S.9)

is a square of the characteristic quasi-particle velocity ($u_{g}^{2}=\frac{5}{3}\left\langle v_{x}^{2} \right\rangle$
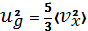
), $E_{av}={e_{g}}/{n_{g}}$ is the average quasi-particle energy in the thermalized region of the quasi-particle spectrum, and $m=\hbar/\left( 2D_{x} \right)$ is the effective quasi-particle’s mass (for anisotropic spectrum – effective mass for acceleration in the $x$ direction). In the case of quasi-particles with finite spectral gap, the average energy $E_{av}$ is measured from the minimum particle’s energy (gapped case can be transformed to gapless form Eq. (S.4) by a simple redefinition of the chemical potential $\mu$). The spin wave dispersion $D_{x}=\left( {\partial^{2}\omega}/{\partial k_{x}^{2}} \right)/2$ near the spectral minimum in the in-plane magnetized YIG film, needed to estimate the effective mass of a magnon $m=1.5\times{10}^{-30} \mathrm{kg}$, as well as the lower boundary of the magnon spectrum $\omega_{min}(k)$ were calculated using the explicit dispersion equations (36), (47) in [S3] (or Eqs. (45), (A-10) in (S-4)).

In the absence of relaxation ($\Gamma_{n,p,e}=0$), Eqs. (S.8) describe the second sound waves having the simple dispersion relation

$\Omega_{K}=u_{g}K$. (S.10)

Thus, $u_{g}$ is the speed of a second sound wave in a *dissipationless* medium. It can be shown that, in the case of an ideal gas, the expression Eq. (S.9) for the sound speed $u_{g}$ coincides with the usual expression $u_{g}^{2}={\partial P}/{\partial\rho}$ ($P$ is the gas pressure, $\rho$ is its density, and the derivative is taken at constant entropy).

As seen from the structure of Eqs. (S8), the second sound waves in a gas of quasi-particles are induced by the oscillations between the fluctuations of the momentum and energy densities, while the fluctuations of the density of quasi-particles passively follow the fluctuations of the linear momentum according to Eq. (S.8a). Therefore, the relaxation parameter $\Gamma_{n}$ does not appear in the equations for the dispersion of the second sound wave.

In the presence of relaxation (i.e. for the case of a non-vanishing contact of the quasi-particle subsystem with the other degrees of freedom) Eqs. (S.8) allow one to find a complex dispersion relation for the dissipative second sound waves in the form:

$\Omega_{K}^{2}+i\left( \Gamma_{p}+\Gamma_{e} \right)\Omega_{K}-\left( \Gamma_{p}\Gamma_{e}+u_{g}^{2}K^{2} \right)=0$. (S.11)

Separating real and imaginary parts of the second sound wavenumber, $K=K^{'}+iK''$, one can obtain a real dispersion relation (see Eq. (3a) in the main text) for the dissipative second sound, and, also, a dependence of the second sound wave damping coefficient $K''$ on the wave's wavenumber $K'$ (see Eq. (3b) in the main text) .

Note, that the dimensionless parameter $q$ in Eqs. (3) of the main text ($q={2\sqrt{\Gamma_{p}\Gamma_{e}}}/\left( \Gamma_{p}+\Gamma_{e} \right)$) depends on the ratio of the momentum and energy relaxation rates. It is equal to $q=1$ for $\Gamma_{p}=\Gamma_{e}$ and $q<1$ for $\Gamma_{p}\neq\Gamma_{e}$. For the case $q=1$ ($\Gamma_{p}=\Gamma_{e}$) the dissipative second sound wave has the same linear dispersion $\Omega_{K}=u_{g}K'$ as in the dissipationless case. The presence of relaxation in this case only leads to a finite damping coefficient $K^{''}=\kappa$ (i.e., finite propagation distance $L=1/{K''}={u_{g}}/{\Gamma_{p}}$ of the second sound wave).

The parameters $q$ and $\kappa$ of the dispersion law Eq. (3) for the magnonic second sound as functions of the pumping power (or density of the magnon gas) were extracted from fitting of the experimental dispersion curves similar to the ones presented in Fig. 3 measured at different values of the pumping power. These curves are presented in Fig. S1 for pumping powers both below and above the threshold of formation of the magnonic BEC at room temperature. It is clear from Fig. S1 that the relaxation asymmetry parameter $q$ has a pronounced minimum at the threshold of the BEC formation, but with the increase of the magnon gas density this asymmetry decreases, and the value of $q$ approaches unity.

In the limiting case $\Gamma_{e}=0$, which corresponds to the usual *phononic* second sound (in the gas of phonons with Umklapp processes) [S5, S6], the parameter $q$ vanishes ($q\to0$). In this case the long-wavelength ($K^{'}\to0$) dispersion of the second sound wave has a *diffusive* character: $\Omega_{K}=\left( {u_{g}}/\kappa\right){K^{'}}^{2}$, $K^{''}=K^{'}$.

For the case of a *magnonic* second sound the relaxation rates $\Gamma_{p}$ and $\Gamma_{e}$ are caused, mainly, by the same physical processes, and, thus, should have the same order of magnitude (i.e., $0<q<1$). Moreover, since the dominant “extrinsic” relaxation process is the scattering to higher-energy (non-thermalized) magnon states, one should expect $\Gamma_{e}>\Gamma_{p}$, since such processes are more intense for magnons that have larger energies, and contribute more to the energy fluctuations rather than to the fluctuations of the number of quasiparticles or their linear momentum.


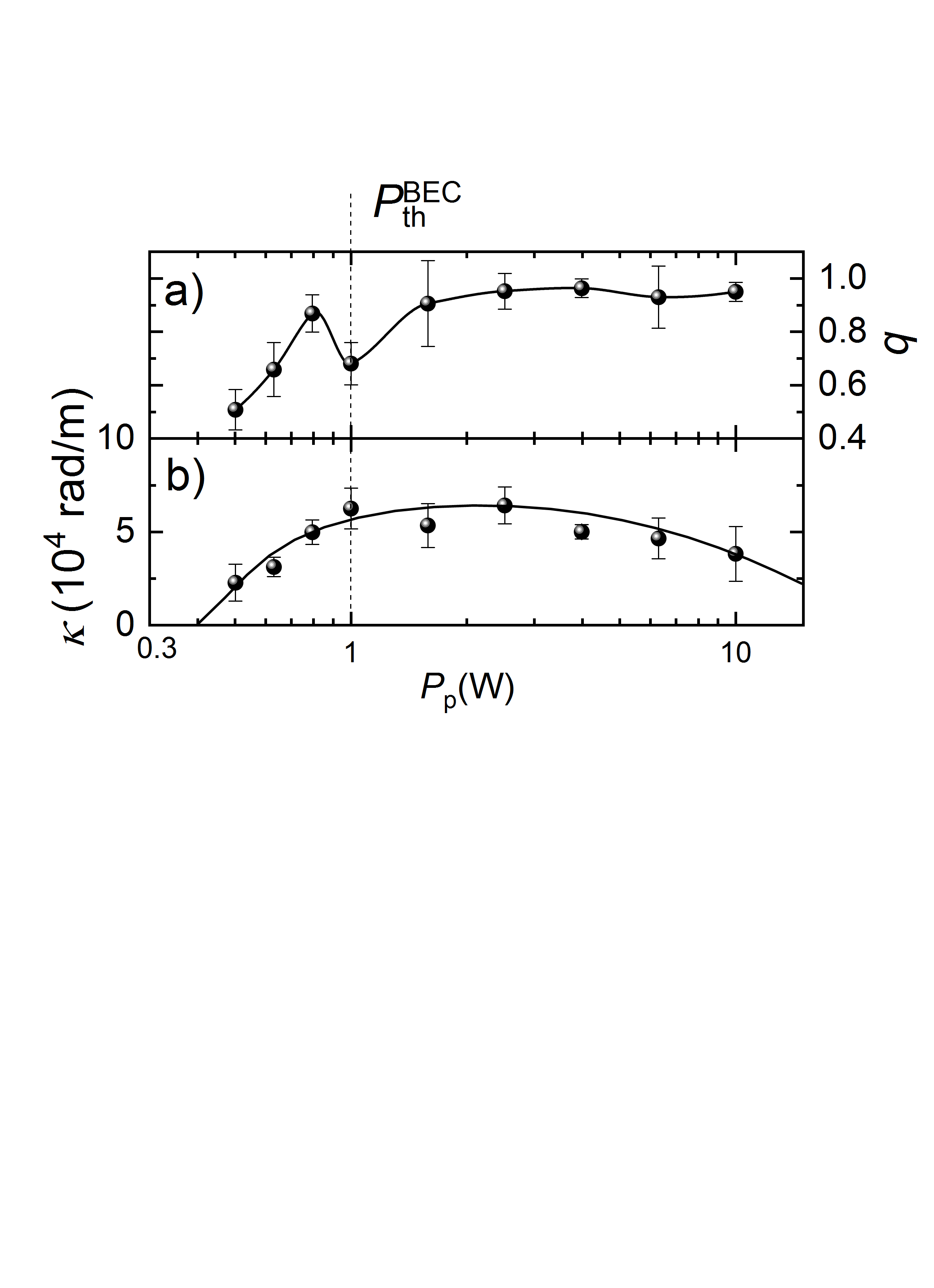


Fig. S.1: (a) Relaxation asymmetry parameter $q={2\sqrt{\Gamma_{p}\Gamma_{e}}}/\left( \Gamma_{p}+\Gamma_{e} \right)$ characterizing the difference between the relaxation rates $\Gamma_{e}$ and $\Gamma_{p}$ for the energy and linear momentum of quasi-particles, respectively, and (b) the characteristic second sound damping parameter $\kappa=\left( \Gamma_{p}+\Gamma_{e} \right)/{2u_{g}}$ vs. the pumping power calculated from the experimental dispersion curves similar to those shown in Fig. 3 by fitting Eqs. (3). Vertical dashed line shows the power threshold the BEC ($P_{th}^{BEC}=1 W$) determined from Fig. 2b.

In the general “magnonic” case ($0<q<1$) the second sound velocity remains *finite* in the long-wavelength region: ${\Omega_{K}}/{K^{'}}=qu_{g}$. Thus, the usual diffusive regime of the second sound propagation is absent in this case. The long-wavelength second sound velocity ($qu_{g}$) is lower than that for the dissipationless second sound waves. For the second sound waves with large wavevectors $K^{'}\gg\kappa$ the second sound velocity increases to that of the dissipationless case, ${\Omega_{K}}/{K^{'}}=u_{g}$.

The above presented results show, that measurements of the second sound dispersion and damping can serve as a powerful tool for the characterization of quasi-particle gasses in open thermodynamic systems. Namely, such measurements allow one to find the relaxation rates for energy $\Gamma_{e}$ and momentum $\Gamma_{p}$, and, also, the average quasi-particle energy $E_{av}$.

In the case of conventional gasses, in which thermal equilibrium state is established over all energy scales, the average particle’s energy $E_{av}$ is determined, mainly, by the gas temperature $T$. The situation is different for the pumped magnon gas that is experimentally studied in the current work.

In this case, the thermal “pumped” equilibrium is established only over a limited range of the magnon energies. In the simplest model, one can assume that the pumped equilibrium exists for magnon energies from the bottom of the magnon spectrum $E_{min}=\hbar\omega_{g}$ (determined by the applied bias field $B_{0}$, $\omega_{g}\approx\gamma B_{0}$ (see [S5, S6] for details), where $\gamma=2\pi\cdot28 GHz/T$ is the modulus of the gyromagnetic ratio) to a certain maximum energy $E_{max}=E_{min}+\Delta E$ [S2]. The maximum energy $E_{max}$ of the thermalized (equilibrated) magnons depends, mainly, on the frequency of parametrically injected magnons $\omega_{p}$. Below, we shall use a simple approximation $E_{max}\approx\hbar\left( \omega_{p}-\Delta\omega_{th} \right)$, where $\Delta\omega_{th}$ is the thermalization frequency interval, i.e., interval of frequencies over which injected non-thermal (parametrically pumped) magnons thermalize due to the intrinsic 4-magnon scattering processes.

Since, for typical experimental conditions $E_{max}\ll k_{B}T$, the Bose-Einstein distribution function Eq. (S.2) can be approximated by the Rayleigh distribution with a finite magnitude of a chemical potential $\mu$:

$n_{\boldsymbol{k}}=\frac{k_{B}T}{\hbar\omega_{\boldsymbol{k}}-\mu}$. (S.12)

Then, one can calculate the total density of magnons $n_{g}$ and density of energy $e_{g}$ by integrating $n_{\boldsymbol{k}}$ and $\hbar\omega_{\boldsymbol{k}}n_{\boldsymbol{k}}$ in the energy range $E_{min}<\hbar\omega_{\boldsymbol{k}}<E_{max}$. Using a quadratic approximation for the dispersion relation Eq. (S.4), these integrals can be found analytically, yielding the following expression for the average quasi-particle energy $E_{av}$ of the magnon gas ($E_{av}$ is measured from the minimum energy $E_{min}$):

$E_{av}=F\left( \xi\right)\Delta E=\left( \frac{1}{3\left[ 1-\sqrt{\xi}\arctan\left( 1/\sqrt{\xi} \right) \right]}-\xi\right)\Delta E$, (S.13)

where $\xi=\left( E_{min}-\mu\right)/{\Delta E}$.

Note, that the average magnon energy $E_{av}$ Eq. (S.13) is independent of the gas energy (temperature $k_{B}T$), which is a characteristic feature of a pumped quasi-equilibrium existing only in the low-energy part of the magnon spectrum $\hbar\omega_{\boldsymbol{k}}\ll k_{B}T$, where the temperature determines the overall population of quasi-particles, but not the shape of the distribution function Eq. (S.12).

The average energy Eq. (S.13) increases with the increase of the energy interval $\Delta E$ of the pumped equilibrium. This conclusion can be directly qualitatively checked experimentally: the energy interval $\Delta E$ can be controlled either by changing the bias magnetic field $B_{0}$ (and, thus, the lowest magnon energy $E_{min}$), or by changing the frequency of the parametrically injected magnons $\omega_{p}$ (which changes the maximum energy $E_{max}\approx\hbar\left( \omega_{p}-\Delta\omega_{th} \right)$). The second sound velocity $\backslash V_{K}\propto\sqrt{E_{av}}$ should increase (decrease) when the bias magnetic field decreases (increases) or when the pumping frequency increases (decreases).

The average energy $E_{av}$ depends on the magnon’s chemical potential $\mu$ (through the function $F\left( \xi\right)$ in Eq. (S.13)), namely, it decreases with the increase of $\mu$. This behavior follows from Eq. (S.12), as the lower-energy states become relatively more populated with the increase of the chemical potential $\mu$, compared to the higher-energy states.

Above the threshold of the BEC formation the chemical potential is fixed at $\mu=E_{min}$. In this case $\xi=0$, $F\left( 0 \right)=1/3$, and Eq. (S.13) gives

$E_{av}^{BEC}=\frac{1}{3} \Delta E$, (S.14)

and the average magnon energy reaches its minimum possible value, determined by the shape of the magnon dispersion relation and the energy of the injected magnons. Thus, Eq. (S.13) gives a simple qualitative explanation of the experimentally observed dependence of the average magnon energy on the pumping power (proportional to the density of magnons) presented in Fig. 4b.

Expressing all the energies in the units of linear frequency (MHz), taking the value of the average magnon energy ${E_{av}^{BEC}}/h=80 \mathrm{MHz}$ from Fig. 4(b) (which was calculated using Eq. (5) from the experimental Fig. 4(a) measured at the bias magnetic field $B_{0}=133 \mathrm{mT}$ and pumping frequency of ${{2\omega}_{p}}/{2\pi}=9.0 \mathrm{GHz}$), and using Eq. (S.14) one can estimate the spectral width $\Delta E$ of the pumped quasi-equilibrium region in the magnon spectrum:

${\Delta E}/h={3E_{av}^{BEC}}/h=240 MHz$. (S.15)

Then, the upper boundary of the thermalized quasi-equilibrium region of the magnon spectrum is ${E_{max}}/h={\omega_{g}}/{2\pi}+{\Delta E}/h\approx4.0 \mathrm{GHz}$ which is $500 \mathrm{MHz}$ below the injected magnon frequency ${\omega_{p}}/{2\pi}=4.5 GHz$ . These calculations show, that the frequency interval of thermalizaiton of injected magnons is, approximately, ${\Delta\omega_{th}}/{2\pi}\approx500 \mathrm{MHz}$.

This estimation of the thermalization interval $\Delta\omega_{th}$ is in good agreement with our measurements of the field dependence of the second sound phase velocity, performed in the interval of bias magnetic fields $120 \mathrm{mT}<B_{0}<135 \mathrm{mT}$ (see Fig. 6 in the main text). These measurements were performed at the maximum pumping power ($P_{p}=10 W$), when the BEC of magnons was formed, and the chemical potential of the magnon gas was fixed at $\mu=\hbar\omega_{g}$. To obtain the theoretical curve shown in Fig. 6, we used Eqs. (S.9), (S.14), and approximation $E_{max}=\hbar\left( \omega_{p}-\Delta\omega_{th} \right)$ with $\Delta\omega_{th}$ being the only fitting parameter of the model. The best fit was obtained for ${\Delta\omega_{th}}/{2\pi}\approx490 \mathrm{MHz}$, which is rather close to the value, ${\Delta\omega_{th}}/{2\pi}\approx500 \mathrm{MHz}$ obtained in the previous set of experiments.

**REFERENCES**

[S1] Kremer, J. M. An Introduction to the Boltzmann Equation and Transport Processes in Gases (Springer-Verlag , Berlin-Heidelberg, 2010).

[S2] Serga, A.A. *et al*. Bose-Einstein condensation in an ultra-hot gas of pumped magnons. *Nat. Commun.* **5**, 3452 (2014).

[S3] Kreisel, A., Sauli, F., Bartosch, L., & Kopietz, P. Microscopic spin-wave theory for yttrium-iron garnet films. *Eur. Phys. J. B* **71**, 59 (2009).

[S4] Kalinikos, B. A. & Slavin, A. N. Theory of dipole-exchange spin wave spectrum for ferromagnetic films with mixed exchange boundary conditions. *J. Phys. C: Solid State Phys.* **19**, 7013 (1986).

[S5] Bargmann, S. Second Sound Waves in Solids in: *Encyclopedia of Thermal Stresses* (ed. Hetnarski, R. B.) (Springer, Dordrecht, 2014).

[S6] Prohofsky, E. & Krumhansl, J. Second-Sound Propagation in Dielectric Solids. Phys. Rev. **133**, A1403-09 (1964).

[S7] Demokritov, S.O. & Demidov, V.E. Micro-Brillouin Light Scattering Spectroscopy of Magnetic Nanostructures*.* *IEEE Trans. Mag.* **44**, 6 (2008).

[S8] Büttner, O. *et al.* Spatial and Spatiotemporal Self-Focusing of Spin Waves in Garnet Films Observed by Space- and Time-Resolved Brillouin Light Scattering. *J. Appl. Phys.* **87**, 5088 (2000).

1. Correspondence and requests should be addressed to V.T. (email: tyberkev@oakland.edu) [↑](#footnote-ref-2)
